# Auditory Profile-Based Hearing Aid Fitting: Self-Reported Benefit for First-Time Hearing Aid Users

**DOI:** 10.3390/audiolres14010017

**Published:** 2024-02-08

**Authors:** Oscar M. Cañete, Gérard Loquet, Raul Sánchez-López, Dan Dupont Hougaard, Rikke Schnack-Petersen, Michael Gaihede, Jesper H. Schmidt, Dorte Hammershøi, Tobias Neher

**Affiliations:** 1 Research Unit for ORL—Head & Neck Surgery and Audiology, Odense University Hospital & University of Southern Denmark, 5230 Odense, Denmark; rikke.schnack-petersen@rsyd.dk (R.S.-P.); jesper.schmidt@rsyd.dk (J.H.S.); tneher@health.sdu.dk (T.N.); 2 Hearing Systems, Department of Health Technology, Technical University of Denmark, 2800 Lyngby, Denmark; raul.sanchezlopez@igdore.org; 3 School of Psychology, The University of Auckland, 28 Park Ave., Grafton, Auckland 1023, New Zealand; 4 Department of Clinical Medicine, Aalborg University, 9920 Aalborg, Denmark; gerard.loquet@unimelb.edu.au (G.L.);; 5 Institute for Globally Distributed Open Research and Education (IGDORE), 2860 Søborg, Denmark; 6 Department of Otorhinolaryngology, Head & Neck Surgery and Audiology, Aalborg University Hospital, 9000 Aalborg, Denmark; mlg@rn.dk; 7 Odense Patient Data Explorative Network (OPEN), Odense University Hospital, 5000 Odense, Denmark; 8 Department of Electronic Systems, Aalborg University, 9220 Aalborg, Denmark; dh@es.aau.dk

**Keywords:** hearing aids, hearing loss, self-report, questionnaires, SSQ12, IOI-HA

## Abstract

*Background:* Although hearing aids (HAs) can compensate for reduced audibility, functional outcomes and benefits vary widely across individuals. As part of the Danish ‘Better hEAring Rehabilitation’ (BEAR) project, four distinct auditory profiles differing in terms of audiometric thresholds and supra-threshold hearing abilities were recently identified. Additionally, profile-specific HA-fitting strategies were proposed. The aim of the current study was to evaluate the self-reported benefit of these profile-based HA fittings in a group of new HA users. *Methods*: A total of 205 hearing-impaired older adults were recruited from two Danish university hospitals. Participants were randomly allocated to one of two treatment groups: (1) NAL-NL2 gain prescription combined with default advanced feature settings (‘reference fitting’) or (2) auditory profile-based fitting with tailored gain prescription and advanced feature settings (‘BEAR fitting’). Two months after treatment, the participants completed the benefit version of the short form of the Speech, Spatial, and Qualities of Hearing Scale (SSQ12-B) and the International Outcome Inventory for Hearing Aids (IOI-HA) questionnaire. *Results:* Overall, participants reported a clear benefit from HA treatment. However, no significant differences in the SSQ12-B or IOI-HA scores between the reference and BEAR fittings were found. *Conclusion*: First-time users experience clear benefits from HA treatment. Auditory profile-based HA fitting warrants further investigation.

## 1. Introduction

Hearing aids (HAs) are the most common and effective treatment for hearing loss [1]. In clinical practice, HA fittings are based on (1) pure-tone audiometric thresholds, (2) the experience of hearing-care professionals, and (3) patient feedback [2,3]. Despite substantial technological advances in the last decades (e.g., with the advent of digital HAs), a significant proportion of patients do not use their HAs, with reported non-use rates ranging from 18% to 57% [4,5]. Factors related to the perceived value (e.g., limited benefits in noisy situations) and physical fit or comfort (e.g., ease of handling) are major reasons for not using HAs [6]. Especially, first-time users (e.g., patients with presbycusis) struggle to adjust to new sounds [4] and to develop new routines necessary for HA management [6], leading to dissatisfaction among many users during the initial stages of HA treatment [7].

The primary purpose of HAs is to compensate for reduced audibility due to hearing loss. However, it has been reported that functional outcomes and benefits (e.g., speech recognition in noise) vary widely among individuals, even in the case of similar hearing thresholds [8]. Since hearing thresholds cannot capture deficits at suprathreshold levels (e.g., impaired temporal processing abilities), inter-individual differences in suprathreshold processing could, to some extent, explain these deficits [9,10].

To address these inter-individual differences, Sanchez-Lopez et al. [11] recently used an auditory test battery combined with a data-driven approach to identify four subgroups of hearing-impaired listeners referred to as profiles A, B, C, and D. In short, profile A is characterized by a mildly sloping high-frequency hearing loss and no or only slight speech intelligibility (SI) and loudness perception (LP) deficits. Profile B is characterized by a sloping high-frequency hearing loss combined with clear SI deficits. Profile C is characterized by pure-tone thresholds >30 dB HL at low (<1 kHz) frequencies and >50 dB HL at high (≥2 kHz) frequencies with SI and LP deficits. Profile D is characterized by a flat hearing loss with pure-tone thresholds >30 dB HL across the audiometric frequencies and LP deficits.

**Figure 1:**
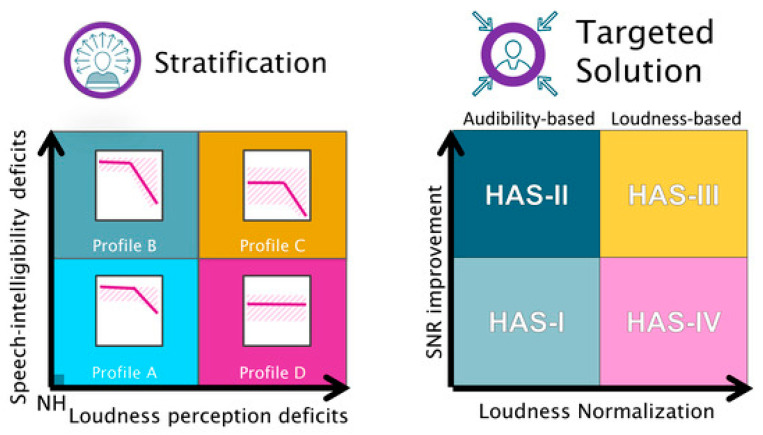
Illustration of the profile-based HA-fitting strategy. (**Left**): Summary of the auditory profiling results. In a two-dimensional space with speech intelligibility (SI)-related deficits on the y-axis and loudness perception (LP)-related deficits on the x-axis, listeners differing in the degree of these two types of deficits are placed at different positions along the two dimensions. (**Right**): Hearing aid settings (HAS) for the different profiles, which are intended to compensate for the specific auditory deficits. Signal-to-noise ratio (SNR) improvement as a solution for SI deficits and loudness normalization as a solution for LP deficits [12].

To build on these findings, Sánchez-López et al. [12] proposed an auditory profile-based fitting rationale to compensate for the deficits observed for the four profiles. This fitting rationale focuses on signal-to-noise (SNR) improvement to compensate for SI deficits, and low-frequency gain prescription to normalize LP (Figure 1; left). Consequently, profiles B and C receive more noise reduction to improve SNR and listening comfort, whereas profiles A and D have noise reduction restricted to acoustically challenging situations. In terms of gain prescription, amplification is exclusively provided in the high frequencies. For profiles C and D, amplification is provided across all frequencies as loudness normalization is targeted (Figure 1; right).

Questionnaires are a well-established means of documenting the subjective benefits from HA intervention [13]. The International Outcome Inventory for Hearing Aids (IOI-HA) [14] and the Speech, Spatial, and Qualities of Hearing Scale (SSQ) [15] are two questionnaires that are widely used in both research and clinical practice for assessing different domains of self-reported benefit [16,17]. The IOI-HA is a questionnaire designed to assess the effectiveness of HA treatment, whereas the SSQ measures auditory disabilities across a wide range of daily-life listening situations. Various SSQ versions are available [18], including a 12-item benefit version for HA users called the SSQ12-B [19]. The IOI-HA and SSQ offer a comprehensive overview of a listener’s daily life experiences following hearing intervention.

Several reports have shown that questionnaires are sensitive to changes in HA fitting and signal processing [20,21]. Anderson et al. [21] used two questionnaires, the SSQ and the Effectiveness of Auditory Rehabilitation [22], to assess differences in signal processing parameters (e.g., in terms of frequency compression and amplitude compression time constants). Their study indicated that both questionnaires were sensitive to capturing such differences (incl. qualities of sound and speech perception in complex environments).

Given that auditory profile-based HA fitting represents a novel approach to hearing rehabilitation, the current study aimed to assess self-reported benefits achievable with this approach with a group of new adult HA users. The new fitting approach was compared with a reference approach consistent with international guidelines. In addition, given the limited available information about the psychometric properties of the SSQ12-B, this study also investigated aspects such as internal consistency, floor and ceiling effects, and the readability of this measure.

## 2. Materials and Methods

The current study was conducted as part of the Danish ‘Better hEAring Rehabilitation’ (BEAR) project. The data were collected at the Departments of Audiology at Odense University Hospital (OUH), Region of Southern Denmark, and at Aalborg University Hospital (AAUH), North Jutland Region.

### 2.1. Participants

A total of 205 adults (mean age: 68.3 ± 7.5 years; range: 45–83 years; 54% male) were recruited from the regular patient flow at the two university hospitals in Aalborg and Odense. The inclusion criteria were as follows: (1) bilateral symmetric (difference in air conduction thresholds at all octave frequencies from 0.5 to 4 kHz ≤15 dB) sensorineural hearing loss, (2) air conduction thresholds ≤50 dB HL at frequencies ≤1 kHz, (3) no evidence of a fluctuating hearing loss over the past 12 months, (4) post-lingual onset of hearing loss, (5) no disabling tinnitus, (6) Danish as primary language, and (7) no prior experience with HAs.

### 2.2. Test Protocol

**Figure 2:**
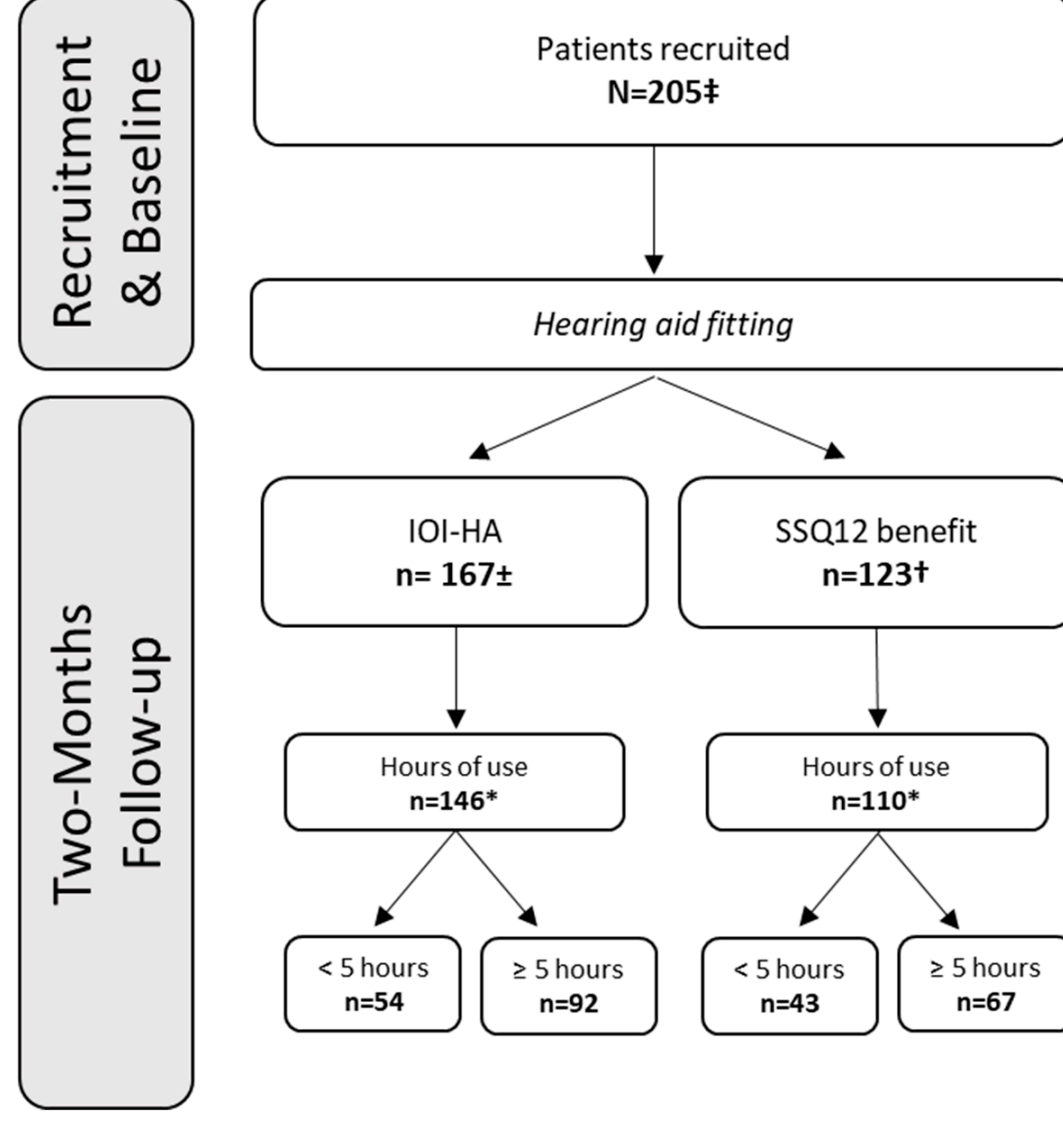
Number of respondents for the IOI-HA and SSQ12-B questionnaires. ±: Mean PTA across 0.5, 1, 2, and 4 kHz = 32.2 dB HL, SD = 6.5 dB HL. †: Mean PTA across 0.5, 1, 2, and 4 kHz = 33.0 dB HL, SD = 7.7 dB HL. *: Data-logging information available. ‡: Reference fitting group, N = 105; BEAR fitting group, N = 100.

The participants attended four visits that were scheduled as follows: (1) hearing examination based on audiometry, tympanometry, and middle ear muscle reflexes, (2) auditory profiling, (3) bilateral HA fitting with real-ear measurements and aided performance tests, and (4) 2-month follow-up with HA adjustments and real-ear measurements (if needed) and aided performance retests (Figure 2).

### 2.3. Auditory Profiling

Auditory profiling was conducted based on the results of the aforementioned test battery and a data-driven approach [23]. The test battery included measures of audibility (e.g., pure-tone audiometry), speech perception in noise [24], binaural processing (e.g., binaural pitch detection), loudness perception (e.g., adaptive categorical loudness scaling) and spectro-temporal resolution [11,25]. Using the data-driven method, the participants were classified into profiles A, B, C, and D, or they were left unclassified (U) [25]. As indicated in Table 1, in terms of the number of cases, profile B was the largest and profile D the smallest subgroup.

### 2.4. HA Fittings

All participants were fitted with two receiver-in-the-ear (RITE) HAs. Oticon Opn S1, GN Resound LinX2, or Widex Enjoy Fusion 440 instruments were used for this purpose. The participants were assigned randomly to one of two HA-fitting groups: the reference fitting group or the auditory profile-based fitting group. For the sake of brevity, the latter will be referred to as the ‘BEAR fitting’ group below. An effort was made to balance the two fitting groups and three HA models. Custom earmolds (N = 152/205) or instant domes (N = 53/205) were used, as appropriate.

The reference fitting included gain prescription according to the ‘National Acoustic Laboratories-Non-Linear 2’ (NAL-NL2) fitting rule [26], which is commonly used for adults. All other HA settings (incl. microphone directionality and noise reduction) directly followed the default recommendations in the fitting software of the respective manufacturers.

The BEAR fittings were based on the auditory profiling results (for details, see Sanchez-Lopez et al. [27]). Unclassifiable participants (*N* = 7) were allocated to the ‘reference fitting’ group. Profiles A and B received high-frequency amplification and fast-acting compression. Profiles C and D received amplification across all frequencies, and slow-acting compression was applied. For profiles A and D, noise reduction was limited to very challenging environments. For profiles B and C, noise reduction was provided throughout. Any other advanced features available in the test devices did not differ from the manufacturers’ default settings. Importantly, only one-third of the participants could be fitted in full adherence to the above guidelines. This was because two of the test devices used here did not allow for the compressor time constants to be adjusted.

All gain targets were verified with an Interacoustics Affinity 2.0 system. Insertion gains were adjusted to be within ±5 dB of the target values from 0.5–4 kHz [28].

### 2.5. Self-Report Measures

Two months after the HA fitting, the participants completed the SSQ12-B and IOI-HA questionnaires. The questionnaires were administered online using the Research Electronic Data Capture system (REDCap) developed by Vanderbilt University, Nashville, Tennessee, United States [29,30], and hosted by the Open Patient Data Explorative Network (OPEN) at Odense University Hospital.

The SSQ12-B [19] items are grouped into three subscales: Speech (speech in quiet, speech in noise, speech in speech, multiple speech streams), Spatial (localization, distance, movement), and Qualities (segregation, identification of the sound, quality and naturalness of sound, listening effort). The SSQ12-B ratings were made using a visual analogue scale. The scale ranged from −5 to +5. A score of -5 indicates that the experience is “much worse” with HAs than without, while a score of +5 indicates that it is “much better”. The scale’s midpoint (0) indicates that the listening experience is unchanged from the baseline.

The IOI-HA contains seven items: (1) use of HAs (‘Use’), (2) perceived benefits (‘Ben’), (3) residual activity limitation (‘RAL’), (4) satisfaction (‘Sat’), (5) residual participation restriction (‘RPR’), (6) impact on others (‘Ioth’), and (7) change in the quality of life (‘QoL’). Each item has five possible choices. The left end represents the worst possible outcome, and the right end represents the best possible outcome. These seven items can be reduced to two underlying factors: (1) factor 1 (“Me and my hearing aids”), which includes Use, Ben, Sat, and QoL, and (2) factor 2 (“Me and the rest of the world”), which includes RAL, RPR, and Ioth [31].

### 2.6. Data Analysis

SPSS statistics version 26.0 (IBM Corporation, Armonk, NY, USA) was used for the data analysis. Only the data from participants with fully completed questionnaires who had used their HAs for at least 5 h/day [21] as determined using HA data-logging during the 2-month follow-up were included.

As mentioned above, the unclassifiable participants (N = 7) were assigned to the reference fitting group. Because this resulted in a skewed distribution, these participants were excluded from the data analyses.

The Shapiro–Wilk test was applied to assess normality for all datasets. Nonparametric tests were used to compare groups and conditions whenever the assumption of normality was not met. Categorical variables (e.g., gender) were analyzed with chi-squared tests. Between-group comparisons of the two fitting strategies were conducted using Mann–Whitney U-tests. Across profiles, comparisons were performed using Kruskal–Wallis tests. Bonferroni correction was used to adjust the alpha level of 0.05 according to the number of paired comparisons performed across tests during post hoc testing.

The internal consistency of the SSQ12-B questionnaire was assessed using Cronbach’s alpha, with α > 0.7 indicating acceptable reliability for clinical purposes [32]. 

## 3. Results

### 3.1. Demographics

As can be seen in Table 2, the participant groups did not differ in terms of mean age, sex, or PTA4 for the two sets of questionnaire scores.

### 3.2. IOI-HA Scores

Table 3 shows that the overall mean IOI-HA score was 4.2, indicating general satisfaction among the participants with their HAs, regardless of fitting strategy. The item-specific mean IOI-HA scores ranged from 3.7 to 4.4, with the highest score for Ioth (item 6) and the lowest score for QoL (item 7). When the two fitting strategies were compared, no significant differences in IOI-HA scores were found (all *p* > 0.08).

**Figure 3:**
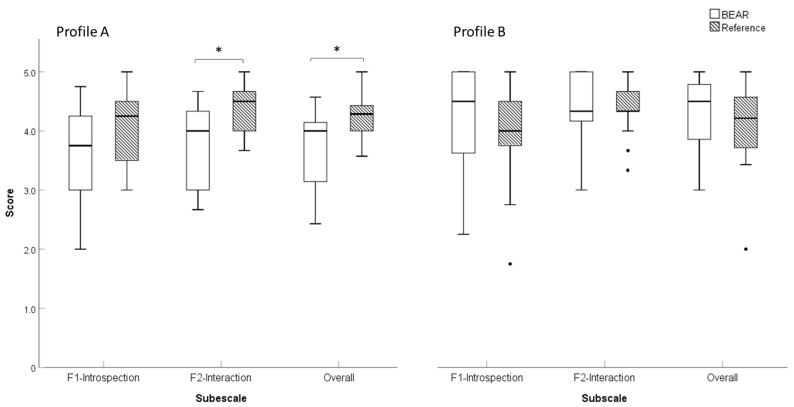
Boxplots of the IOI-HA scores for profiles (**A**,**B**) and the two fitting strategies (BEAR and reference). Profile (**A**): BEAR (*N* = 13), reference (*N* = 14). Profile (**B**): BEAR (*N* = 28), reference (*N* = 28). Asterisks represent statistically significant differences (*p* < 0.05).

Given the substantial imbalance in terms of the size of the auditory profiles (A = 27, B = 56, C = 4, D = 3), statistical comparisons of the two fitting strategies were only made for profiles A and B (Figure 1). Among participants classified as profile A, differences were found for Factor 2 (Interaction) scores (U = 36.5, *p* = 0.013, *d* = 1.1) and overall IOI-HA scores (U = 42.5, *p* = 0.032, *d* = 0.9), with the participants in the reference fitting group reporting more benefit. For profile B, no differences were observed (Figure 3).

### 3.3. SSQ12-B Scores

The SSQ12-B scores showed an HA benefit, regardless of fitting strategy (Table 4). The most significant improvement was seen for the Speech subscale. However, no differences between the two fitting strategies were found (all *p* > 0.05). The reference fitting group showed slightly higher (better) scores than the BEAR fitting group in terms of the overall SSQ scores and SSQ subscales (Speech, Spatial, and Qualities). The BEAR fitting group reported the poorest (lowest) scores for the Spatial subscale.

**Figure 4:**
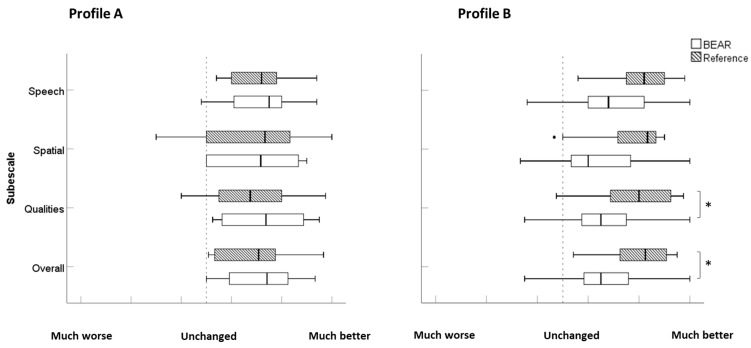
Boxplots of SSQ12-B scores for profiles (**A**,**B**) and the reference and BEAR fittings. Profile (**A**): BEAR (*N* = 12), reference (*N* = 9). Profile (**B**): BEAR (*N* = 21), reference (*N* = 19). Asterisks represent statistically significant differences (*p* < 0.05).

Again, because of the unbalanced group sizes (A = 21, B = 40, C = 3, D = 1, U = 2), the two fitting strategies were only compared for profiles A and B. No significant differences were observed (all *p* > 0.05). As illustrated in Figure 4, the profile B participants in the reference fitting group reported a greater benefit than those in the BEAR fitting group in terms of Qualities (U = 116.5, *p* = 0.024, d = 0.13) and overall benefit (U = 118.5, *p* = 0.028, d = 0.12) scores. Notably, for the Speech and Spatial subscales, there was a benefit for the reference fitting (U = 127.5, *p* = 0.051, d = 0.64, and U = 128.0, *p* = 0.052, *d* = 0.64, respectively).

### 3.4. SSQ12-B Properties

#### 3.4.1. Internal Consistency

The reliability analysis encompassing all 12 items for the total sample (*N* = 123) showed a Cronbach’s alpha of 0.95, signifying high internal consistency, and a Gutmann split-half correlation of 0.93, with alpha coefficients of 0.94 for part 1 (items 1–6) and 0.91 for part 2 (items 7–12). Cronbach’s alpha was also calculated for each of the three subscales (Speech, Spatial, and Qualities). All values were above the criterion threshold of 0.7 for clinical acceptability (Speech: 0.94, Spatial: 0.95, Qualities: 0.87).

Item-total correlations ranged from 0.68 (item 12) to 0.86 (item 7), with a mean inter-item correlation of 0.79. Spearman’s correlation coefficient for the SSQ domain and overall scores ranged from 0.73 (Spatial vs. Qualities) to 0.94 (Speech vs. Overall).

#### 3.4.2. Floor Effects, Ceiling Effects, and Missing Data

None of the participants gave the lowest possible score (−5 points), and only 0.8% gave the highest possible score (5 points) averaged across all 12 items. Hence, floor and ceiling effects were minimal. The “not applicable” option was categorized as missing data. Missing data among the 164 participants who completed the SSQ12-B questionnaire ranged from 0% (item 12) to 14.6% (item 4).

#### 3.4.3. Readability Analysis

The readability of the SSQ12-B was evaluated using the ‘Læsbarhedsindex’ (LIX) [33], a validated index for assessing the readability of written information in Danish. The LIX classifies text into five categories based on the obtained scores: very easy (<24), easy (25–34), standard/medium readability (35–44), difficult (45–54), and very difficult (>55) [34]. The number of words per item varied from 10 to 30 (mean = 21.9 words), totaling 261 words. Individual item scores ranged from 17 (item 3) to 32 (items 2 and 5) points. 

The LIX analysis gave an overall score of 25 points for the SSQ12-B. Qualities, with 27 points, was the most challenging subscale, followed by Speech with 25 points and Spatial with 24 points. Overall, this suggests that the Danish SSQ12-B is easy to read for general audiences (e.g., readers of weekly magazines or fiction) [35].

## 4. Discussion

The current study showed that first-time users perceive clear benefits from HA treatment. However, no differences were observed between the two fitting strategies (reference vs. BEAR). The lack of differences is evident from the IOI-HA scores, which showed similar patterns for the two fitting strategies (Table 1). Item 7 (quality of life) scores were lower than item 6 (impact on others) scores, indicating a positive effect of HA use, for example, in terms of improving communication with others. These findings are similar to previously reported IOI-HA results for Danish first-time HA users [36]. 

For the SSQ12-B scores, no significant differences were observed between the two fitting strategies either—neither for the overall scores nor for any subscale. As for the IOI-HA scores, the participants reported an overall benefit at the group level (Table 2). A clear benefit was reported for clarity of everyday sounds (item 11), while less benefit was observed in terms of the ability to segregate complex sounds (item 9).

The profile A participants in the reference fitting group reported greater benefits in terms of IOI-HA F2 and overall IOI-HA scores relative to those in the BEAR fitting group. The IOI-HA F2 is related to interactions with others (“Me and the rest of the world”), which can be taken as evidence that the reference fitting group experienced fewer difficulties with daily-life activities and communication than the BEAR fitting group. However, no significant differences were observed between the groups in terms of IOI-HA F1 (introspection) scores.

The SSQ12-B results differ from the IOI-HA results when comparisons are made within profiles but between fitting groups. These disparities may be attributed, to some extent, to differences in the ability of the questionnaires to capture differences between HA features. Notably, profile B participants in the reference fitting group reported greater benefits across SSQ12-B subscales and in terms of the overall score (higher scores). The most pronounced difference was seen for the Qualities subscale, where a benefit was reported for situations related to sound segregation, sound identification, qualities and naturalness, and listening effort [18]. Also, there was a trend for the Speech and Spatial subscales to present higher scores within the reference fitting group for profile B participants. The SSQ12-B results indicated that profile A participants did not report significantly different benefits, regardless of the fitting strategy. In contrast, profile B participants with the reference fitting showed significant benefits as compared to the BEAR fitting across the SSQ12-B subscales. These differences could be related to the BEAR fitting for profile B, for example, more high-frequency gain and more aggressive NR compared to the reference fitting, potentially influencing perceived sound quality.

Sanchez-Lopez et al. [27] hinted at a potential risk of the BEAR fittings being perceived as inferior to the reference fitting. For profile B participants, aggressive NR and fast-acting compression were applied and compared to the reference fitting (with standard NR and amplitude compression settings). This could have impacted sound naturalness and speech clarity, especially for new HA users. In other words, the limited amount of NR provided to profile B participants in the reference fitting group could have positively impacted the outcome for these individuals. Besides, profile A participants treated with the BEAR fittings did not have any directionality or noise suppression activated.

Regarding gain prescription, the HAs for both groups were fitted to target using real-ear measurements, thereby effectively providing more gain than what is typically prescribed for first-time HA users in the Danish clinical population [37]. It cannot be ruled out that this factor by itself or in combination with others (e.g., NR and directionality) had a negative impact on aided outcome. That said, this factor cannot explain the (lack of) differences between profiles A and B or between the BEAR and reference fittings, as the same approach to gain prescription was followed for all participants.

### 4.1. Differences across Fitting Strategies

The BEAR fittings for profiles A and B are designed to maximize audibility, amplifying high frequencies to enhance speech perception, as explained by Sanchez-Lopez et al. [12]. In the current study, the use of directionality and NR was limited; that is, an omnidirectional microphone setting and ‘mild’ NR were used [12]. In the present study, this was accomplished by providing slightly more gain to high frequencies relative to NAL-NL2, fasting-acting compression to achieve audibility benefits, and NR for SNR improvement. However, as mentioned above, only one-third of the participants were fitted in complete adherence to these guidelines. Therefore, the potential benefits of the BEAR fitting strategy may have been reduced because of technical constraints in some test devices. Overall, the differences between the reference and BEAR fittings could have been rather small for these two profiles, despite efforts to increase the contrast between the two strategies.

While the reference and BEAR fittings were closely aligned in terms of gain prescription, the default settings by different manufacturers could have introduced features that positively impacted self-perceived benefits, particularly for the group that received the reference fittings. For example, variations across NR algorithms (e.g., less aggressive settings), adaptive directionality, or additional noise management features could have influenced the listening experiences of the reference fitting group. Notably, these types of features were either unavailable or limited in the BEAR fittings, potentially explaining the observed variability in self-perceived benefits for this group, as evident in the SSQ12-B scores.

It is also important to note that the effects of HA features such as NR and microphone directionality can strongly depend on the acoustic environments they are used in. For instance, activation of such features may vary across devices and manufacturers. Additionally, if the main benefit from this approach is expected in noisy environments and the patient spends most of their time in quiet environments, they may not perceive an improvement. These types of differences could also have impacted the results of the current study by overshadowing self-perceived benefits.

### 4.2. Differences across Outcome Measures

The two outcome measures used here (IOI-HA and SSQ12-B) can be assumed to be sensitive to capturing differences between HA fittings, so any differences in reported benefit could be traced back to the specific purpose of each questionnaire. The IOI-HA provides a general overview of HA satisfaction [31], whereas the SSQ12 provides information about listening abilities in different daily-life contexts [15]. Broadly speaking, the differences between the IOI-HA and SSQ12-B scores observed here support this view. On the one hand, the profile A listeners fitted with the reference strategy reported significantly higher IOI-HA scores, but these differences were not present in the SSQ12-B scores. On the other hand, the profile B participants fitted with the BEAR strategy showed a trend for more benefit in terms of the IOI-HA scores. However, the SSQ12-B scores showed the opposite pattern, their scores being lower than those of the participants fitted with the reference strategy.

Finally, for the online version of the SSQ12-B used here, our analyses demonstrated good internal consistency for both the overall scores and the three subscales. This is consistent with findings for other language versions of the SSQ12-B, for example, the Turkish version (Speech: 0.86; Spatial: 0.76; Qualities: 0.81 [38]), indicating good consistency for all subscales of this questionnaire. Additionally, negligible floor and ceiling effects were observed, along with good readability. These results indicate that the tool is reliable for assessing self-perceived hearing abilities in hearing aid users.

### 4.3. Limitations and Further Research

Several limitations should be acknowledged. First, the profile subgroups were clearly unbalanced in terms of size, with about 80% of the participants belonging to profiles A and B. These two profiles are characterized by relatively good suprathreshold hearing abilities that are entangled with reduced audibility at high frequencies (see Section 1). This could perhaps explain why no differences between the reference and BEAR fittings were found for these subgroups, as both fitting strategies are based on maximizing speech audibility.

It is also possible that, acoustically speaking, the two fitting strategies were too similar for any differences to emerge. In future work, the use of another study design that facilitates the assessment of such HA conditions (e.g., a crossover design) could help reveal benefits from profile-based HA fitting.

Future work would also need to address the shortage of profile C and D participants observed here. It appears that, relative to profiles A and B, prevalence is lower for these subgroups in the population of first-time users with bilateral hearing loss. Thus, more time would need to be allocated to the recruitment of sufficiently large profile C and D subgroups, or the inclusion criteria must be adjusted so that the benefits from profile-based HA fitting can be investigated properly.

## 5. Conclusions

The primary objective of the current study was to investigate the self-reported benefits from auditory profile-based HA fitting in first-time users in a clinical setting. The auditory profile-based (BEAR) fittings were meant to better compensate for individual differences in hearing thresholds and suprathreshold hearing abilities, as compared with an audiogram-based (reference) approach. The results showed a clear overall benefit from HA provision. However, no discernible differences between the two fitting approaches were found. Further research into auditory profile-based HA fitting with evenly balanced participant subgroups is warranted, so that potential user benefits can be investigated properly.

## Figures and Tables

**Table 1:** Distribution of IOI-HA and SSQ12-B respondents according to profile and fitting strategy.

Questionnaire		HA Fitting	
IOI-HA	*N* = 167/205 (81.6%)	Reference	BEAR
A	52	27	25
B	89	44	45
C	12	7	5
D	7	3	4
U *	7	7	
SSQ12-B			
Profile	*N* = 123/205 (60.0%)		
A	41	18	23
B	62	31	31
C	10	5	5
D	8	3	5
U *	5	5	

* U: Unclassified.

**Table 2:** Participant demographics.

	SSQ12-B			IOI-HA		
	Reference Fitting (*N* = 30)	BEAR Fitting (*N* = 37)	*p*-Value	Reference Fitting (*N* = 50)	BEAR Fitting (*N* = 42)	*p*-Value
Mean age in years (SD)	66.3 (7.7)	68.6 (7.2)	0.269	69.1 (7.0)	66.7 (7.3)	0.181
Sex						
Male	17	22	0.179	27	23	0.351
Female	13	15		23	19	
Mean PTA4 in dB HL (SD)	31.5 (5.6)	32.1 (6.9)	0.729	33.3 (7.0)	31.0 (5.6)	0.132

**Table 3:** Mean IOI-HA scores (and standard deviations) for the two HA-fitting strategies.

		HA Fitting		
Item	*N* = 92	Reference (*N* = 50)	BEAR (*N* = 42)	*p*-Value
1 (F1-Use)	4.4 (0.8)	4.3 (0.8)	4.4 (0.8)	0.474
2 (F1-Ben)	3.9 (1.0)	3.8 (0.9)	4.1 (1.1)	0.081
3 (F2-RAL)	3.9 (0.8)	4.0 (0.7)	3.8 (1.0)	0.271
4 (F1-Sat)	4.2 (1.0)	4.2 (1.0)	4.2 (1.0)	0.898
5 (RPR)	4.3 (0.9)	4.4 (0.8)	4.2 (1.0)	0.568
6 (F2-Ioth)	4.7 (0.7)	4.7 (0.7)	4.6 (0.6)	0.086
7 (F1-Qol)	3.7 (0.9)	3.7 (0.9)	3.7 (1.0)	0.824
F1–introspection	4.0 (0.8)	4.0 (0.8)	4.1 (0.8)	0.379
F2–interaction	4.3 (0.6)	4.4 (0.5)	4.2 (0.7)	0.305
Overall	4.2 (0.6)	4.2 (0.6)	4.1 (0.7)	0.838

Use: Hearing aid use. Ben: Hearing aid benefit. RAL: Residual activity limitation. Sat: Satisfaction. RPR: Residual participation restriction. Ioth: Impact on others. QoL: Quality of life. Note: Average HA use ranged from 5.0 to 16.0 h/day (mean: 9.2 h/day, SD: 3.0 h/day). The *p*-values reflect the significance of the pairwise comparisons.

**Table 4:** Mean SSQ12-B scores (and standard deviations) for the two fitting strategies.

		HA Fitting		
Items	*N* = 67	Reference (*N* = 30)	BEAR (*N* = 37)	*p*-Value
1	2.6 (1.7)	2.8 (1.7)	2.4 (1.8)	0.264
2	2.1 (1.8)	2.2 (1.9)	1.9 (1.8)	0.376
3	2.5 (1.6)	2.7 (1.5)	2.4 (1.6)	0.309
4	2.5 (1.6)	2.7 (1.5)	2.3 (1.8)	0.321
5	2.6 (1.7)	2.9 (1.3)	2.4 (1.9)	0.307
Speech	2.4 (1.5)	2.7 (1.4)	2.3 (1.6)	0.300
6	2.1 (1.9)	2.3 (1.9)	1.9 (1.8)	0.202
7	1.9 (1.7)	2.2 (1.6)	1.7 (1.8)	0.319
8	2.1 (1.9)	2.4 (2.0)	1.9 (1.9)	0.285
Spatial	2.0 (1.7)	2.3 (1.7)	1.8 (1.7)	0.368
9	1.7 (1.9)	1.9 (2.1)	1.6 (1.7)	0.393
10	2.0 (2.0)	2.4 (2.1)	1.7 (1.8)	0.085
11	2.7 (2.0)	2.9 (2.1)	2.6 (1.9)	0.319
12	2.3 (1.9)	2.7 (1.3)	2.1 (2.2)	0.336
Qualities	2.2 (1.6)	2.5 (1.6)	2.0 (1.6)	0.211
Overall	2.3 (1.5)	2.5 (1.5)	2.1 (1.5)	0.264

Note: Average HA use ranged from 5.0 to 16.0 h/day (mean: 8.9 h/day, SD: 3.0 h/day).

## Data Availability

The data presented in this article are available from the corresponding author upon reasonable request.
